# Outcome predictors of odontogenic abscesses in the elderly

**DOI:** 10.3389/froh.2024.1486182

**Published:** 2024-12-02

**Authors:** Daniel Kaercher, Philipp Thelen, Mike Ruettermann, Lei Li, Axel Hamprecht

**Affiliations:** ^1^ Department for Oral and Maxillofacial Surgery, Klinikum Oldenburg AöR, Oldenburg, Germany; ^2^ Institute of Medical Microbiology and Virology, University of Oldenburg and Klinikum Oldenburg AöR, Oldenburg, Germany; ^3^ HPC Oldenburg—Institute for Hand and Plastic Surgery, Oldenburg, Germany; ^4^ University Medical Center Groningen—UMCG, University of Groningen, Groningen, Netherlands

**Keywords:** odontogenic abscess, elderly, geriatric patients, chronic renal failure, C-reactive protein

## Abstract

Odontogenic infections have a high prevalence and can lead to severe complications. Due to demographic changes, the number of geriatric patients has increased in recent years. The aim of this study was to analyse odontogenic abscesses in elderly patients and to differentiate them from non–elderly patients regarding clinical presentation, bacterial analysis and therapy. We retrospectively reviewed 1,173 inpatients with odontogenic abscesses from 2014 to 2020. Patients were divided into elderly patients (≥70 years, *n* = 240) and non-elderly patients (<70 years, *n* = 933). Demographics, clinical parameters, laboratory values and treatment parameters were analysed. Overall, elderly patients had a longer hospital stay (LOS) (median 4 [range 28] vs. 3 [range 22] days) and more complications (9.6% vs. 7.9%) than non-elderly patients, although these differences were not statistically significant. Peri-/submandibular (*p* = 0.015), parapharyngeal (*p* < 0.001) and oral base infections (*p* = 0.036) were associated with significantly longer LOS in the elderly. Chronic renal failure (CRF) was associated with LOS (*p* = 0.010) and complications (*p* = 0.006). In the elderly, c-reactive protein (CRP) correlated significantly with LOS (*p* < 0.001) and more complications (*p* = 0.036). This study identifies anatomical spaces and CRF as outcome predictors of odontogenic abscesses in the elderly. In addition, CRP level may serve as a predictor of complicated course in elderly patients.

## Introduction

Infections of the oral and maxillofacial region have a high incidence and can cause severe complications (1–3), such as mediastinitis, airway obstruction or sepsis (4, 5).

Odontogenic infections are the most common cause of oral and maxillofacial abscesses (2, 6–8).

Several general and local predisposing factors are known to increase the risk of severe odontogenic infections. These include for example unstable diabetes mellitus (DM), immunosuppression, history of radiation and/or chemotherapy (9–21).

Orofacial odontogenic infections are frequently mixed aerobic and anaerobic infections (6, 22). The bacterial spectrum is diverse, and the microbiology result often excogitates the existence of commensal oral flora (6, 23).

Treatment of odontogenic infection is based on surgical drainage, focus remediation and antibiotic therapy (6, 24, 25). However, if the abscess is treated incorrectly or late, the infection may spread in deeper facial spaces, where it becomes difficult to treat and may be fatal (26–29). In addition to surgery, the choice of empirical antibiotics is also critical (26, 30). Widespread indifferent use of antibiotics has led to the emergence of resistant bacteria (31).

According to the joint definition of the German Society of Geriatric Medicine (DGG), the German Society of Gerontology and Geriatrics (DGGG), the Federal Association of Clinical geriatric Institutions (BAG) and the Professional Association of German Internists Section geriatrics, the geriatric patient is characterised by advanced age, mostly 70 years and older, combined with a geriatric–typical multimorbidity (32–34). Elderly patients are considered high-risk patients with a higher probability of prolonged hospitalisation (33, 35).

Odontogenic infections affect people of all ages (28). However, age-associated decline in reserve and function may reduce the ability to cope with acute external stressors, typically defined as frailty (36, 37). Frailty is associated with a higher risk of poor outcomes such as disability and mortality (38). Inflammation may be closely associated with frailty (39). In the elderly, their reduced immunological reaction and comorbidities may be associated with an increase in severe infections (40). In addition, diagnosis and treatment of odontogenic infections in the elderly may be more complex than in younger patients because of more comorbid conditions (41).

The number of individuals aged 60 and over worldwide is expected to increase to more than 2 billion by 2050 (42).

Thus, there will be more elderly patients with oral and maxillofacial infections (1). Only a few studies consider age as an important factor in head and neck infections, but without specification in odontogenic infections (1, 3).

We aimed to analyse the differences in clinical features, treatment modalities, and bacterial analysis of odontogenic infections between elderly and non-elderly patients. We hypothesise that we will identify variables associated with worse outcome to improve the clinical risk stratification of elderly patients.

## Material and methods

### Study design

All patients with upper and lower jaw infections requiring admission to our hospital between January 2014 and April 2020 were included in this retrospective study. Patients with International Statistical Classification of Diseases and Related Health Problems (ICD) codes K 10.3, K10.2, K 12.2, K 14.0 were primarily included. ICD-criteria were fulfilled by 2,902 patients.

With reference to the valid definition of the DGG, the DGGG and other professional societies, we divided the patients into two subgroups: older than or equal to 70 years (*n* = 711) and younger than 70 years (*n* = 2,191).

To study only infections with an odontogenic focus, exclusion criteria were: Patients with antiresorptive-associated necrosis of the jaw (ARONJ), or patients with an antiresorptive medication (*n* = 311). Furthermore, in order to achieve improved comparability the results, we excluded patients with head and neck cancer (*n* = 146), infection without a dental cause (*n* = 192), patients who had received radiation therapy to the head and neck (*n* = 19), primary osteomyelitis (*n* = 32), foreign body infections (*n* = 30), patients who had undergone tooth extraction without a surgical incision and drainage (*n* = 99) and patients who had received antibiotic therapy without surgical intervention (*n* = 80). Furthermore, we excluded outpatients (*n* = 819) because of insufficient data. Overall, a total of 1,173 patients (elderly *n* = 240 patients and non-elderly *n* = 933 patients) were included.

The institutional ethics committee of the Carl von Ossietzky University Oldenburg approved the study.

### Data collection

Data were collected from the clinical information system (CGM Medico, Release 27.01.04.01, CGM Clinical Europe GmbH, Koblenz, Germany).

All patients underwent a full diagnostic workup, including medical history, physical examination, laboratory tests and radiological imaging.

To obtain further information, we analysed the doctor's letter, premedication, operative report, medication list, laboratory values, microbiological report, radiological imaging and clinical history of each patient.

Factors included in the analysis were age, sex, selected systemic diseases, dental aetiology, treatment modalities, leukocytes, C-reactive protein levels, microbiology results and living situation.

### Selected systemic diseases

Based on a literature review, DM, immunosuppression, history of malignancy or chemotherapy, cardiovascular disease, chronic respiratory disease, CRF [GFR < 30 ml/min, stage G4 Kidney Disease: Improving Global Outcome Classification (KDIGO)], dementia, cerebrovascular disease were chosen as relevant systemic diseases.

### Laboratory values

The White blood cell count (WBC) in Tsd./*μ*l and CRP in g/dl, taken at the time of first contact with the emergency department were analysed.

### Surgery

All patients received surgical abscess incisions and intravenous antibiotic treatment (except eight patients) with different antibiotics. We included patients with intraoral and extraoral incisions or both. The sample for microbiological pathogens and resistance was obtained from the first purulent secretion that appeared. The submandibular approach was chosen as standard for drainage of larger mandibular abscesses. For other sites (e.g., temporal or in the cheek), an appropriately established surgical approach was chosen.

### Microbiological analysis

After incision and access to the abscess pocket swabs were taken if deemed necessary by the surgeon. To avoid contamination, samples were taken directly from the abscess secretion carefully avoiding any contact to the surrounding tissue. However, possible unconscious or unintentional contamination cannot be ruled out completely due to the often small surgical incision, especially in case of intraoral incisions. Samples were inoculated on BD Columbia agar + 5% sheep blood, BD Chocolate agar, BD Schaedler agar and BD Schaedler KV agar (all BD, Heidelberg, Germany). Columbia and chocolate agar were incubated for 48 h at 35 ± 2°C and 5% CO2 atmosphere. Schaedler agar and Schaedler KV agar were incubated for 72 h at 35 ± 2°C under anaerobic conditions.

Plates were read after 24, 48 and 72 h. Bacteria that were considered relevant were further identified (Table 2). No growth or a mixture of bacteria of the common oral flora was considered as “no growth of pathogenic bacteria”.

Identification of bacteria was performed biochemically with the Vitek 2 system (bioMérieux, Nuertingen, Germany) or with MALDI-TOF MS using the MALDI Biotyper microflex system (Bruker Daltonics, Bremen, Germany). Susceptibility testing results were excluded from the analysis due to inconsistent testing strategies during the study period.

### Statistics

After pseudonymisation, a database was constructed using Microsoft Excel (Version 16.59, Microsoft, Redmond, WA, USA).

Continuous variables were presented as median and range [in the tables also as mean ± standard deviation (SD)] and analysed using the Mann–Whitney *U*-test. Categorial variables were evaluated using the Chi–squared test.

Regression analysis was also performed. First univariate linear regression analysis was used for numerical outcomes and univariate binary logistic regression for categorial outcomes.

Thereafter, statistically significant and historically reported important variables were analysed by multivariate regression analysis.

Biometric advice was provided by the Institute of Biometrics and Clinical Research Münster, Germany.

All statistical analyses were performed with SPSS version 21 (SPSS, Chicago, Illinois). A *p*-value < 0.05 was considered statistically significant.

## Results

### Patients and clinical data

We analysed 1,173 patients with odontogenic infections. Of these 240 (20.5%) were elderly patients with an age equal to or greater than 70 years and 933 (79.5%) were non-elderly patients with an age less than 70 years.

Five hundred forty-nine (46.8%) were females and 624 (53.2%) males. The median age of all patients was 48.79 [range 77.08] years. In the elderly, median age was 78.98 [range 25.25] years. In the non-elderly the median age was 41.92 [range 51.64] years.

A total of 1,131 patients (96.4%) lived in their own home, 42 patients (3.6%) in a nursing home. In the elderly, 30 patients (12.5%) lived in a nursing home compared to only 12 non-elderly patients (1.3%), (*p* < 0.001).

### Systemic diseases

Overall, the most common co-morbidity was cardiovascular disease (*n* = 170; 14.5%), followed by DM (*n* = 99; 8.4%) and chronic respiratory disease (*n* = 84; 7.2%). In the elderly, the most common co–morbidity was also cardiovascular disease, (*n* = 97; 40.4%), followed by DM, (*n* = 47; 19.6%), and cerebrovascular disease, (*n* = 37; 15.4%).

There was a significant difference between the elderly and non-elderly groups in all systemic diseases examined, except for immunosuppressive medication (Table 1).

**Table 1:** Baseline and clinical data of the patients.

	All patients	Elderly patients	Non-elderly patients	*p*-value
Gender				
Female	549 (46.8%)	130 (54.2%)	419 (44.9%)	0.011
Male	624 (53.2%)	110 (45.8%)	514 (55.1%)	0.011
Age (years)				
Mean ± SD	50.01 ± 19.99	79.51 ± 6.03	42.42 ± 14.54	<0.001
Median [range]	48.79 [77.08]	78.98 [25.25]	41.92 [51.64]	
Living situation				
Own house	1,131 (96.4%)	210 (87.5%)	921 (98.7%)	<0.001
Nursing home	42 (3.6%)	30 (12.5%)	12 (1.3%)	<0.001
Systemic diseases				
Cardiovascular diseases	170 (14.5%)	97 (40.4%)	73 (7.8%)	<0.001
Diabetes mellitus	99 (8.4%)	47 (19.6%)	52 (5.6%)	<0.001
Chronic respiratory diseases	84 (7.2%)	25 (10.4%)	59 (6,3%)	0.035
Cerebrovascular diseases	59 (5.0%)	37 (15.4%)	22 (2.4%)	<0.001
Malignant diseases	52 (4.4%)	26 (10.8%)	26 (2.8%)	<0.001
Dementia	48 (4.1%)	28 (11.7%)	20 (2.1%)	<0.001
Chronic renal failure	33 (2.8%)	28 (11.7%)	5 (0.5%)	<0.001
Immunosuppressive medication	35 (3.0%)	10 (4.2%)	25 (2.7%)	0.285
Infection space				
Peri/submandibular	395 (33.7%)	49 (20.4%)	346 (37.1%)	<0.001
Paramandibular/vestibular	342 (29.2%)	100 (41.7%)	242 (25.9%)	<0.001
Fossa canina	130 (11.1%)	35 (14.6%)	95 (10.2%)	0.064
Mouth base	91 (7.8%)	20 (8.3%)	71 (7.6%)	0.686
Parapharyngeal space	89 (7.6%)	6 (2.5%)	83 (8.9%)	<0.001
Maxillary/Palate	70 (6.0%)	18 (7.5%)	52 (5.6%)	0.284
Cheek	53 (4.5%)	11 (4.6%)	42 (4.5%)	0.957
Causative tooth				
Lower molars	544 (46.4%)	66 (27.5%)	478 (51.2%)	<0.001
Lower premolars	136 (11.6%)	44 (18.3%)	92 (9.9%)	<0.001
Lower front and canines	69 (5.9%)	41 (17.1%)	28 (3.0%)	<0.001
Upper molars	54 (4.6%)	11 (4.6%)	43 (4.6%)	0.987
Upper premolars	48 (4.1%)	10 (4.2%)	38 (4.1%)	0.948
Upper front and canines	95 (8.1%)	31 (12.9%)	64 (6.9%)	0.003
Infection after tooth extraction	227 (19.4%)	37 (15.4%)	190 (20.4%)	0.099
Incision site				
Intraoral incision	617 (52.6%)	167 (69.6%)	450 (48.2%)	<0.001
Extraoral incision	500 (42.6%)	61 (25.4%)	439 (47.1%)	<0.001
Combined intra—and extraoral incision	56 (4.8%)	12 (5.0%)	44 (4.7%)	0.865
Laboratory values				
WBC: *n* = 798; elderly: *n* = 170, non-elderly: *n* = 628 CRP: *n* = 710; elderly: *n* = 145, non-elderly: *n* = 565 *mean (median)* *±* *SD [range]*				
White blood cell count (Tsd./µl)	12.37 (11.80) ± 4.62 [32.3]	11.05 (10.35) ± 4.35 [24.1]	12.73 (12.25) ± 4.63 [32.3]	<0.001
c-reactive protein (g/dl)	9.43 (6.80) ± 8.90 [57.4]	8.47 (6.30) ± 8.62 [43.8]	9.67 (7.00) ± 8.96 [57.0]	0.064

### Infection space

Overall, the peri/submandibular lodge was most frequently involved (*n* = 395; 33.7%), followed by the paramandibular/vestibular space, (*n* = 342; 29.2%) and the fossa canina space (*n* = 130; 11.1%). Older patients had significantly more infections in the paramandibular/vestibular space and in the fossa canina space. In contrast younger patients had significantly more infections in the peri/submandibular and parapharyngeal area (Table 1).

### Causative teeth

The most causative tooth for infection was one of the lower molars, (*n* = 544; 46.4%). Two hundred twenty-seven patients (19.4%) had an infection after tooth extraction, 115 patients (50.7%) after extraction of wisdom teeth and 112 (49.3%) after extraction of other teeth.

In younger patients, an infection after tooth extraction was the second leading cause of odontogenic abscess (*n* = 190; 20.4%) (Table 1).

### Surgery

In general, 617 patients (52.6%) had an intraoral incision and 500 patients (42.6%) an extraoral incision. Fifty-six patients (4.8%) had a combined incision (Table 1).

### Laboratory values

Leukocyte count was performed on admission in 798 patients, CRP was determined in 710 patients. The median value of WBC was 11.80 [range 32.3] Tsd./μl, the median value of CRP was 6.80 [range 57.4] g/dl (Table 1).

### Microbiological analysis

Microbiological analysis was performed in 616 patients (52.5%). No pathogenic bacteria were found in *n* = 203 (33.0%); in the remaining 413 cases, 81 different species were found, which we grouped into 14 bacterial groups.

Overall, the most frequently isolated bacteria were members of the *Streptococcus anginosus* group (*S. anginosus, S.constellatus and S. intermedius*), (*n* = 146; 23.7%), followed by *Prevotella* species (spp.) in 135 patients (21.9%) and coagulase negative staphylococci (CNS), (*n* = 110; 17.9%).

In older patients, the most frequently isolated bacteria also belong to the *Streptococcus anginosus* group, (*n* = 19; 20.7%), followed by *Prevotella* spp., (*n* = 17; 18.5%) and CNS, (*n* = 15; 16.3%). This order can also be observed in the younger patients.

The only significant difference in isolation frequency between younger and older patients was found in the viridans group streptococci (*n* = 6/75; *p* = 0.044) and *Candida* spp. (*n* = 9/15; *p* = 0.005) (Table 2).

**Table 2:** Spectrum of bacteria groups isolated in the patients (*p < 0.05).

	All patients	Elderly patients	Non-elderly patients	*p*-value
Microbiological diagnostic performed	616 (52.5%)	92 (38.3%)	524 (56.2%)	
Bacterial isolates *(n* *=* *616 patients, elderly* *=* *92, non-elderly* *=* *524)*				
No pathogenic bacteria isolated	203 (33.0%)	36 (39.1%)	167 (31.9%)	0.339
Strep. anginosus spp.	146 (23.7%)	19 (20.7%)	127 (24.4%)	0.456
Prevotella spp.	135 (21.9%)	17 (18.5%)	118 (22.5%)	0.388
Coagulase negative Staphylococci (CNS)	110 (17.9%)	15 (16.3%)	95 (18.1%)	0.673
Viridans streptococci	81 (13.1%)	6 (6.5%)	75 (14.3%)	0.044*
Porphyrmonas/Bacteroides spp.	48 (7.8%)	6 (6.5%)	42 (8.0%)	0.622
Enterobacteales (gram negative)	29 (4.7%)	4 (4.3%)	25 (4.8%)	0.860
Candida spp.	24 (3.9%)	9 (9.8%)	15 (2.9%)	0.005*
Other anaerobic germs	21 (3.4%)	2 (2.2%)	19 (3.6%)	0.479
Fastidious gram-negative rods of the oral flora	15 (2.4%)	1 (1.1%)	14 (2.7%)	0.363
Staphylococcus aureus	14 (2,3%)	3 (3.3%)	11 (2.1%)	0.490
Parvimonas micra	12 (1.9%)	3 (3.3%)	9 (1.7%)	0.323
Other bacteria	7 (1.1%)	0 (0.0%)	7 (1.3%)	0.270
Fusobacterium nucleatum	6 (1.0%)	2 (2.2%)	4 (0.8%)	0.204
A,B,C,G—Streptococci	4 (0.6%)	1 (1.1%)	3 (0.6%)	0.571

### Antimicrobial treatment

Eight patients (0.7%) received no antibiotic treatment. Eight hundred and eighty-six patients (75.5%) received ampicillin/sulbactam (Unacid®) and 161 patients (13.7%) received clindamycin according to local and national guidelines (43). In addition, 85 patients (7.2%) received a combination of ampicillin/sulbactam and metronidazole and ten patients (0.9%) received a combination of clindamycin and metronidazole.

### Complications

Overall, 97 patients (8.3%) had complications. Elderly patients had more complications, (*n* = 23; 9.6%) than non-elderly patients, (*n* = 74; 7.9%), (*p* = 0.407).

The most frequently complication was intensive care treatment (*n* = 60; 5.1%), mainly due to upper airway obstruction, (*n* = 38/60; 63.3%).

Furthermore, ICU admission in cause of upper airway obstruction mainly occured in patients with infection in the large and deep spaces, like the parapharyngeal or perimandibular/submandibular space.

Younger patients were more frequently admitted to an intensive care unit (ICU) (*p* = 0.038), also regarding to upper airway obstruction (*p* = 0.029).

Furthermore, patients with an abscess in the parapharyngeal space were significantly more admitted to the ICU (*p* = 0.001) due to upper airway obstruction (*p* < 0.001) (Table 3).

**Table 3:** Complications in the patients (*p < 0.05).

	All patients	Elderly patients	Non-elderly patients	*p*-value
Complication present *(n* *=* *97 patients)*				
	97 (8,3%)	23 (9,6%)	74 (7,9%)	0,407
Complications in detail *(n* *=* *150 complications, multiple complications/patient possible)*				
ICU therapy	60 (5.1%)	10 (4.2%)	50 (5.4%)	0,038*
Upper airway obstruction	38 (3.2%)	3 (1.3%)	35 (3.8%)	0,051
Sepsis	5 (0.4%)	2 (0.8%)	3 (0.3%)	0,278
Necrotizing fasciitis	2 (0.2%)	-/-	2 (0.2%)	0,473
Mediastinitis	1 (0.1%)	1 (0.4%)	-/-	0,049*
Chronic Osteomyelitis	8 (0.7%)	3 (1.3%)	5 (0.5%)	0,231
Second surgery	38 (3.2%)	12 (5.0%)	26 (2.8%)	0,084
Complications in ICU patients *(n* *=* *60 patients, elderly* *=* *10/non-elderly* *=* *50)*				
Upper airway obstruction	38 (63.3%)	3 (30.0%)	35 (70.0%)	0.029*
Sepsis	5 (8.3%)	2 (20.0%)	3 (6.0%)	0.144
Necrotizing fasciitis	1 (1.6%)	0 (0.0%)	1 (2.0%)	0.652
Mediastinitis	1 (1.6%)	1 (10.0%)	0 (0.0%)	0.024*
Chronic osteomyelitis	1 (1.6%)	0 (0.0%)	1 (2.0%)	0.652
Second surgery	9 (15.0%)	2 (20.0%)	7 (14.0%)	0.628
Intensive Care therapy for other reasons	5 (8.3%)	-/-	-/-	-/-
ICU admission and upper airway obstruction in relation to infection site				
Infection site	ICU admission (*n* = 60)		Upper airway obstruction (*n* = 38)	
	Patients	*p*-value	Patients	*p*-value
Peri/submandibular	26 (43.3%)	0.787	16 (42.1%)	0.979
Paramandibular/vestibular	0 (0.0%)	0.001	0 (0.0%)	0.042
Fossa canina	1 (1.7%)	0.302	0 (0.0%)	0.158
Mouth base	10 (16.7%)	0.777	6 (15.8%)	0.718
Parapharyngeal space	18 (30.0%)	0.001*	15 (39.5%)	<0.001
Maxillary/Palate	1 (1.7%)	0.430	1 (2.6%)	0.210
Cheek	2 (3.3%)	0.051	0 (0.0%)	0.018
Different space	2 (3.3%)	—	0 (0.0%)	—

Performing binary logistic regression analysis, the following parameters showed a significant impact on complications: pre-existing CRF (Odds ratio (OR):3.14 [CI:1.325–7.430], *p* = 0.009), parapharyngeal infection [OR:3,50 (CI:2.006–6.110), *p* < 0.001], base of the mouth infection [OR:2.88 (CI:1.620–5.109), *p* < 0.001] and origin of the infection in the lower molars [OR:1.72 (CI:1.130–2.628), *p* = 0.011].

In elderly patients, the impact of CRF [OR:4.06 (CI:1.501–10.996), *p* = 0.006] on complications increases.

Performing multivariate regression analysis, infection in the perimandibular (OR 1.01 [CI:0.977–1.024], *p* < 0.001, oral base [OR:6.65 (CI:3.012–14.701), *p* < 0.001] and parapharyngeal space [OR:7.16 (CI:3.121–16.432), *p* < 0.001] had a significant impact on complications.

Regression analysis also showed that the likelihood of complications increases with a higher WBC [OR:1.10 (CI:1.054–1.150), *p* < 0.001] and CRP [OR:1.08 (CI:1.057–1.104), *p* < 0.001], however the multivariate regression analysis showed only a significant prognostic impact of CRP [OR:1.06 (CI1.034–1.088), *p* < 0.001] (Table 4).

**Table 4:** Binary logistic regression analysis for complications (*p < 0.05), (*RC, regression coefficient, OR, odds ratio, CI, 95% confidence interval*).

Univariate logistic regression analysis								
Parameter	All patients				Elderly			
	RC	OR	CI	*p*-value	RC	OR	CI	*p*-value
age	0.01	1.01	0.994-1.015	0.366	—	—	—	—
Systemic diseases								
Cardiovascular diseases	−0.10	0.91	0.493-1.664	0.750	−0.48	0.62	0.244-1.562	0.309
Diabetes mellitus	−0.03	0.97	0.457–2.069	0.943	−1.79	0.17	0.022–1.287	0.086
Chronic respiratory diseases	0.57	1.76	0.898–3.438	0.100	0.68	1.97	0.611–6.321	0.257
Cerebrovascular diseases	−0.54	0.58	0.178–1.891	0.367	−0.21	0.81	0.227–2.868	0.741
Malignant diseases	−0.40	0.67	0.205–2.187	0.506	0.24	1.27	0.349–4.588	0.720
Dementia	0.48	1.62	0.671–3.917	0.283	0.85	2.34	0.795–6.906	0.123
Chronic renal failure	1.14	3.14	1.325–7.430	0.009*	1.40	4.06	1.501–10.996	0.006*
Immunosuppressive medication	0.37	1.21	0.501–4.196	0.493	−19.01	0.00	0.000–-/-	0.999
Infection space								
Peri/submandibular	0.40	1.49	0.979–2.278	0.063	1.26	3.51	1.436–8.586	0.006*
Paramandibular/vestibular	−1.93	0.09	0.063–0.335	<0.001*	−1.33	0.27	0.087–0.806	0.019*
Fossa canina	−1.43	0.24	0.074–0.764	0.016*	−19.13	0.00	0.000–/-	0.998
Mouth base	1.06	2.88	1.620–5.109	<0.001*	0.97	2.65	0.803–8.714	0.110
Parapharyngeal space	1.25	3.50	2.006–6.110	<0.001*	1.62	5.07	0.876–29.347	0.070
Maxillary/Palate	−1.88	0.15	0.021–1.107	0.063	−19.05	0.00	0.000–-/-	0.998
Cheek	0.72	2.06	0.942–4.504	0.070	0.79	2.20	0.446–10.864	0.333
Causative tooth								
Lower molars	0.54	1.72	1.130–2.628	0.011*	1.19	3.29	1.374–7.892	0.008*
Lower premolars	−0.94	0.39	0.156–0.982	0.046*	−0.92	0.40	0.090–1.759	0.224
Lower front and canines	0.06	1.06	0.447–2.516	0.895	−0.35	0.71	0.200–2.498	0.590
Upper molars	−0.88	0.42	0.099–1.729	0.227	−19.01	0.00	0.000–-/-	0.999
Upper premolars	−0.31	0.73	0.223–2.398	0.605	−19.01	0.00	0.000–-/-	0.999
Upper front and canines	−2.22	0.11	0.015–0.789	0.028*	−1.26	0.28	0.037–2.181	0.226
Infection after tooth extraction	0.28	1.33	0.812–2.173	0.258	0.47	1.61	0.557–4.633	0.381
Laboratory values								
White blood cell count (Tsd./µl)	0.10	1.10	1.054–1.150	<0.001*	0.10	1.10	0.996–1.218	0.060
c-reactive protein (g/dl)	0.08	1.08	1.057–1.104	<0.001*	0.05	1.05	1.003–1.103	0.036*
Multivariate logistic regression analysis								
Independent variable	All patients							
	RC		OR		CI		*p*-value	
age	0.01		1.01		0.977–1.024		0.126	
Diabetes mellitus	0.28		1.32		0.555–3.149		0.529	
Chronic renal failure	0.29		1.34		0.338–5.284		0.679	
Perimandibular/submandibular space	1.20		3.32		1.699–6.492		<0.001*	
Mouth base space	1.895		6.65		3.012–14.701		<0.001*	
Parapharyngeal space	1.969		7.16		3.121–16.432		<0.001*	
White blood cell count (Tsd./µl)	0.004		1.00		0.949–1.063		0.881	
c-reactive protein (g/dl)	0.059		1.06		1.034–1.088		< 0.001	

Performing ROC-analysis we also saw a higher diagnostic accuracy for CRP (Area under the curve (AUC):0.692 [CI:0.625–0.758], *p* < 0.001) than WBC [AUC: 0.611 (CI:0.541–0.681), *p* = 0.001] (Table 5).

**Table 5:** ROC analysis for WBC and CRP as predictor for complications (*p < 0.05), (*AUC, area under the curve, CI, 95% confidence interval*).

Laboratory value	All patients			Elderly			Non - elderly		
	AUC	CI	*p*-value	AUC	CI	*p*-value	AUC	CI	*p*-value
White blood cell count (Tsd./µl)	0.611	0.541–0.681	0.001*	0.568	0.427–0.709	0.361	0.628	0.550–0.706	0.001*
C – reactive protein (g/dl)	0.692	0.625–0.758	<0.001*	0.636	0.481–0.790	0.069	0.709	0.636–0.782	<0.001*

### Length of hospital stay

The median hospital stay time was 3 [range 28] days.

Elderly patients had a longer LOS 4 [range 28] days than non-elderly patients 3 [range 22] days, (*p* = 0.129).

Elderly patients with an infection in the peri/submandibular (*p* = 0.038), parapharyngeal (*p* = 0.018) oral base (*p* = 0.017) and cheek space (*p* = 0.004) as well as elderly patients with post-extraction infections (*p* = 0.001) had a significantly more extended LOS than non-elderly patients (Table 6).

**Table 6:** Length of hospital stay (*p < 0.05).

	All patients	Elderly patients	Non-elderly patients	*p*-value
Length of hospital stay (LOS) mean (median) ± SD [range] in days				
	3.92 (3) ± 2.69 [28]	4.27 (4) ± 3.41 [28]	3.84 (3) ± 2.47 [22]	0.129
LOS in relation to systemic diseases Mean (median) ± SD [range] in days				
Cardiovascular diseases	3.98 (3) ± 2.91 [16]	3.99 (3) ± 2.80 [12]	3.97 (3) ± 3.07 [16]	0.640
Diabetes mellitus	4.09 (4) ± 2.93 [14]	3.72 (4) ± 2.70 [12]	4.42 (4) ± 3.11 [14]	0.299
Chronic respiratory diseases	4.32 (4) ± 2.84 [16]	3.88 (4) ± 1.54 [7]	4.51 (4) ± 3.23 [16]	0.792
Cerebrovascular diseases	4.19 (4) ± 2.37 [12]	4.41 (4) ± 2.58 [12]	3.82 (4) ± 1.97 [7]	0.521
Malignant diseases	3.63 (3) ± 2.50 [12]	4.00 (3) ± 3.18 [12]	3.27 (3) ± 1.54 [6]	0.758
Dementia	3.73 (3) ± 3.81 [25]	4.39 (3) ± 4,66 [25]	2.80 (2,5) ± 1.85 [6]	0.109
Chronic renal failure	5.36 (4) ± 5,52 [28]	5.82 (4,5) ± 5.86 [28]	2,80 (3) ± 1.48 [4]	0.214
Immunosuppressive medication	4.46 (3) ± 3.46 [14]	4.70 (4,5) ± 3.56 [14]	4.36 (3) ± 3.49 [14]	0.653
LOS in relation to infection space *Mean (median)* *±* *SD [range] in days*				
Peri/submandibular	4.58 (4) ± 2.53 [22]	5.33 (4) ± 3.37 [20]	4.48 (4) ± 2.38 [22]	0.038*
Paramandibular/vestibular	2.96 (3) ± 1.66 [12]	3.36 (3) ± 2.08 [12]	2.80 (3) ± 1.42 [9]	0.041*
Fossa canina	2.74 (2) ± 1.69 [11]	3.00 (3) ± 1.37 [5]	2.64 (2) ± 1.80 [11]	0.053
Mouth base	4.85 (4) ± 3.05 [16]	5.80 (6) ± 2.55 [10]	4.58 (4) ± 3.14 [16]	0.017*
Parapharyngeal space	5.78 (4) ± 4.16 [28]	11.17 (7.5) ± 9.24 [25]	5.39 (4) ± 3.33 [17]	0.018*
Maxillary/Palate	2.71 (2) ± 1.87 [10]	2.61 (2) ± 1.88 [5]	2.75 (2) ± 1.89 [10]	0.589
Cheek	4.40 (4) ± 2.49 [11]	6.00 (5) ± 2.37 [9]	3.98 (3) ± 2.37 [11]	0.004*
LOS in relation to causative tooth *Mean (median)* *±* *SD [range] in days*				
Lower molars	4.24 (4) ± 3.01 [28]	5.55 (4) ± 5.28 [28]	4.06 (4) ± 2.50 [22]	0.057
Lower premolars	3.43 (3) ± 2.29 [16]	3.55 (3) ± 2.02 [8]	3.37 (3) ± 2.41 [16]	0.436
Lower front and canines	3.84 (4) ± 2.18 [11]	4.38 (4) ± 3.64 [8]	3.83 (3) ± 2.47 [11]	0.906
Upper molars	3.15 (2) ± 2.40 [10]	2.55 (2) ± 1.70 [4]	3.30 (2) ± 2.54 [10]	0.436
Upper premolars	2.79 (2) ± 1.96 [11]	2.60 (3) ± 1.08 [3]	2.84 (2) ± 2.14 [11]	0.755
Upper front and canines	2.55 (2) ± 1.43 [7]	2.97 (3) ± 1.64 [7]	2.34 (2) ± 1.28 [6]	0.068
Infection after tooth extraction	4.49 (4) ± 2.52 [14]	5.49 (5) ± 2.49 [10]	4.30 (4) ± 2.48 [14]	0.001*

Univariate linear regression analysis showed that patient age had a small effect on LOS [RC:0.02 (CI:0.008–0.023), *p* < 0.001].

Regarding systemic diseases, patients with a CRF had a significantly longer LOS [RC:1.48 (CI:0.552–2.411), *p* = 0.002] with a higher impact in older patients [RC:1.76 (CI: 0.420–3.099), *p* = 0.010].

Peri/submandibular [RC:0.99 (CI:0.671–1.314), *p* < 0.001], parapharyngeal [RC:2.00 (CI:1.432–2.574), *p* < 0.001] and oral base [RC:1.00 (CI:0.426–1.573), *p* = 0.001] infections had a significant impact on LOS, again with an increasing impact in the elderly.

In the regression analysis, a higher WBC [RC:0.08 (CI:0.038–0.125), *p* < 0.001] and CRP-level [RC:0.12 (CI:0.094–0.140), *p* < 0.001] at the time of admission leads to a significantly longer LOS. For CRP, these effect increases in older patients [RC:0.21 (CI:0.142–0.278), *p* < 0.001].

Performing multivariate regression analysis pre-existing CRF [RC:1.507 (CI:0.304–2.709), *p* = 0.014], infections in the peri/submandibular [RC:1.364 (CI:0.890–1.838), *p* < 0.001], parapharyngeal [RC:2.783 (CI:2.046–3.520), *p* < 0.001] and oral base space [RC:1.655 (CI:0.929–2.381), *p* < 0.001] are significant predictors for longer LOS. In elderly patients, infections in the parapharyngeal space [RC:6.074 (CI:2.203–9.945), *p* = 0.002] and oral base space [RC:1.958 (CI:0.106–3.810), *p* = 0038] showed a significant association with prolonged LOS.

Furthermore CRP is a significant prognostic marker for longer LOS [RC:0.092 (CI:0.066–0.117), *p* < 0.001] with an increasing impact in the elderly [RC:0.166 (CI:0.081–0.251), *p* < 0.001] (Table 7).

**Table 7:** Linear regression analysis for LOS (*p < 0.05), (*RC, regression coefficient, CI, 95% confidence interval*).

Univariate regression analysis						
Parameter	All patients			Elderly		
	RC	CI	*p*-value	RC	CI	*p*-value
Age	0.02	0.008–0.023	<0.001*	—	—	—
Systemic diseases						
Cardiovascular diseases	0.07	−0.370−0.506	0.760	−0.47	−1.349–0.420	0.302
Diabetes mellitus	0.18	0.373–0.737	0.520	−0.68	−1.768–0.417	0.224
Chronic respiratory diseases	0.43	−0.170-1.026	0.160	−0.43	−1.855–0.991	0.551
Cerebrovascular diseases	0.27	−0.432-0.979	0.447	0.16	−1.040–1.368	0.789
Malignant diseases	−0.30	−1.052-0.446	0.428	−0.30	−1.698–1.100	0.674
Dementia	−0.20	−0.982-0.576	0.609	0.14	−1.212–1.498	0.836
Chronic renal failure	1.48	0.552–2.411	0.002*	1.76	0.420–3.099	0.010*
Immunosuppressive medication	0.55	−0.357–1.455	0.234	0.45	−1.724–2.628	0.683
Infection space						
Peri/submandibular	0.99	0.671–1.314	<0.001*	1.33	0.266–2.397	0.015*
Paramandibular/vestibular	−1.36	−1.688 to −1.028	<0.001*	−1.55	−2.414 to −0.695	<0.001*
Fossa canina	−1.33	−1.819 to −0.848	<0.001*	−1.48	−2.701 to −0.265	0.017*
Mouth base	1.00	0.426–1.573	0.001*	1.67	0.113–3.232	0.036*
Parapharyngeal space	2.00	1.432–2.574	<0.001*	7.08	4.441–9.712	<0.001*
Maxillary/Palate	−1.29	−1.934 to −0.640	<0.001*	−1.79	−3.425 to −0.154	0.032*
Cheek	0.49	−0.248–1.236	0.191	1.73	−0.250–3.884	0.085
Causative tooth						
Lower molars	0.59	0.280–0.895	<0.001*	1.76	0.816–2.712	<0.001*
Lower premolars	−0.56	−1.044 to −0.082	0.022*	−0.88	−2.002–0.235	0.121
Lower front and canines	−0.09	−0.744–0.567	0.791	−0.65	−1.798–0.508	0.271
Upper molars	−0.81	−1.548 to −0.079	0.030*	−1.80	−3.871–0.263	0.087
Upper premolars	−1.18	−1.956 to −0.0405	0.003*	−1.74	−3.905–0.426	0.115
Upper front and canines	−1.50	−2.057 to −0.939	<0.001*	−1.49	−2.775 to −0.209	0.023*
Infection after tooth extraction	0.71	0.318–1.904	<0.001*	1.44	0.252–2.633	0.018*
Laboratory values						
White blood cell count (Tsd./µl)	0.08	0.038–0.125	<0.001*	0.13	−0.004–0.262	0.058
C – reactive protein (g/dl)	0.12	0.094–0.140	<0.001*	0.21	0.142–0.278	<0.001*
Multivariate linear Regression analysis						
Independent variables	All patients			Elderly		
	RC	CI	*p*-value	RC	CI	*p*-value
Age	0.026	0.015–0.037	<0.001*	—	—	—
Diabetes mellitus	0.258	−0.456–0.972	0.478	0.198	−1.355–1.752	0.801
Chronic renal failure	1.507	0.304–2.709	0.014*	1.643	−0.149–3.436	0.072
Perimandibular/submandibular space	1.364	0.890–1.838	<0.001*	1.457	−0.002–2.917	0.050
Mouth base space	1.655	0.929–2.381	<0.001*	1.958	0.106–3.810	0.038*
Parapharyngeal space	2.783	2.046–3.520	<0.001*	6.074	2.203–9.945	0.002*
White blood cell count (Tsd./µl)	−0.030	−0.080–0.019	0.231	−0.105	−0.256–0.046	0.171
C – reactive protein (g/dl)	0.092	0.066–0,.117	<0.001*	0.166	0.081–0.251	<0.001*

## Discussion

The mean age of all patients in our study was 50.0 ± 19.9 years, similar to other studies (7, 44, 45). Also similarly, the incidence of deep neck infections was higher in the elderly (41, 44, 46).

Equal to other studies, males were predominant (53.2%), with a ratio of 1,13:1 (10, 47–49),. In the present study, older patients were predominantly female (54.2%), similar to the study of Chi et al. (41), but in contrast to the findings by Zheng et al. (1).

We found a significant association between CRF and complications. Furthermore, regression analysis showed that CRF was the strongest predictor for complications and a prolonged LOS.

Patients with CRF, especially in end-stage renal diseases (ESRD), have been reported to have a higher risk of infectious complications (50, 51) due to multifactorial mechanisms such as neutrophil dysfunction, uremic toxicity, biological incompatibility, anaemia, iron overload and a dialysis access and procedure (52–54). As described by Dalrymple et al., infection is the second leading cause of death in patients with an ESRD (54). The authors also found that patients with CRF have longer LOS for infection-related admissions than those without (54, 55).

Several factors such as advanced age, high burden of co-morbidities, hypoalbuminemia (56, 57), immunosuppressive therapy (58), nephrotic syndrome (59), uraemia, anaemia or malnutrition (60, 61) may additionally increase the risk of infection in patients with CRF.

We identified two studies that investigated ESRD in relation to head and neck infections.

Chang et al. found a higher incidence rate and cumulative incidence in patients with ESRD than in those without. They also described a higher mortality rate and poor survival in patients with ESRD, although LOS was not significantly longer (62). A limitation is that only a quarter of patients underwent surgical treatment, whereas in our study all patients did. Another limitation is the definition of ESRD. Chang et al. defined the target group based on ESRD-related ICD-9 without specific GFR, whereas our study defined CRF as GFR < 30 ml/min (62).

Tsai et al. reported that patients with deep neck infections and ESRD had a significantly longer LOS, more ICU admissions and a higher mortality rate than patients without. They also found a higher incidence of *methicillin-resistant Staphylococcus aureus* (MRSA) in the ERSD group, which was not investigated in this study (63).

Regarding the top, the studies by Chang et al. and Tsai et al. looked at all types of deep neck infections without specifying odontogenic origin. However, Chang et al. mentioned the odontogenic origin as the most common cause of head and neck infections (62).

Both studies examined the age of the patients in a subanalysis. Tsai et al. described a higher incidence of ESRD in patients younger than 65 years, whereas Chang et al. described no difference between those younger and older than 65 years (62, 63). Both findings contrast with our study, in which older patients had a significantly higher incidence of CRF than non-elderly patients.

Many studies also reported an association between DM and a complicated course of treatment for head and neck infections up to life—threatening complications (11, 64–66).

However, we found no significant correlations between DM and complications or prolonged LOS.

In this study, the most common site of infection in all patients was the peri/submandibular space, which is similar to many other studies (18, 19, 49, 66).

Contrary to the expected accelerated spreading of the infection in compromised elderly patients, these group had significantly more infections in the paramandibular/vestibular, fossa canina or maxillary spaces. Since the study excluded outpatients, we suspect that the number of younger patients with infections in this space may not necessarily be lower but was not included the analysis due to the criteria.

Flynn et al. described these cavities as “low-risk spaces”, with a low incidence of complications (67). Nevertheless, these patients were admitted to the hospital in the present study. We hypothesise that hospitalisation was due to pre-existing systemic disease and possible concomitant (anticoagulant) medication.

Chi et al. reported the parapharyngeal cavity as the most affected space, in older as well as in younger patients with a cut-off age of 65 years. However, this study also included non—odontogenic causes (41).

The lower molar was the most common focus of infection, which is consistent with many other studies (19, 22, 44, 49, 68, 69). Particularly in younger patients, the lower molars were by far the most common focus (51.2%), resulting in many infections in the submandibular space.

In older patients, the distribution of the odontogenic focus was more even. Since the apex of the tooth of origin determines the path of dissemination (22), the results are consistent with the distribution of infection space in the older group.

In our study, infections at the base of the mouth (OR:2.88; *p* < 0.001) and in the parapharyngeal cavity (OR:3.50; *p* < 0.001) lead to significantly more complications. In older patients, infections in the peri/submandibular space significantly increase the likelihood of complications (OR:3.51; *p* = 0.006).

In addition, infections in the lower molars lead to a significantly higher risk of complications (OR:1.72, *p* = 0.011). These results are in line with the studies of Alotaibi et al. and Ylijoki et al. (9, 10).

Due to modern diagnostics and therapy, serious complications after odontogenic infection have a low incidence and mainly occur in the presence of predisposing factors (14, 19, 70).

In our study 97 patients (8.3%) experienced complications, the most commonly of which were ICU admission (5.1%) mainly for upper airway obstruction (3.2%). The percentage of patients requiring ICU admission was lower than previously reported (18, 66, 71, 72). For example, Barber et al. described a percentage of 21.4% requiring ICU admission, with no significant difference in age (21). Gams et al. even reported an incidence of ICU admission up to 45% (66).

Severe odontogenic infections are known to cause upper airway obstruction (1, 8). Adovica et al. reported a complication rate of 11.4%, mainly caused by upper airway obstruction (18). Suehara et al. even reported a complication rate of 50.3%, mainly caused by airway compromise (72).

As widely expected, and in conclusion with our results, the risk of upper airway obstruction depends on the infection space which in turn often depends on the dental origin. Branstetter et al. described the “mylohyoid line” as a significant border in the communication between the mouth base or sublingual space and the submandibular space of the neck. Furthermore, they described that infections with their dental origin in the anterior part of the mandible first affect the sublingual space, whereas infections from the second or third molar can directly spread into the deeper lodges (73). Nevertheless, both spaces communicate via the unattached posterior margin of the mylohyoid muscle (74).

One possible explanation for this quantitative difference in complications and upper airway obstruction is the inclusion of infection sites in some study analyses. For example, infections in the submandibular and parapharyngeal spaces are much more common and require intubation and intensive care therapy to protect the airway.

In this study, 38 patients (3.2%) underwent a second operation to drain all collections, which is consistent with the findings of Gholami et al. (69). The rate was higher in the elderly (5.0%) compared to the non-elderly (2.8%), but not significantly different. Adovica et al. reported a significantly higher reoperation rate in elderly patients compared to younger patients (18).

In the present study the rate of complications was slightly higher in older patients (9.6% vs. 7.9%), but the difference was not significant. Similarly, regression analysis didn't show a significant effect of age on complications. This contrasts with other studies. Zhang et al. reported a higher incidence of life-threatening complications in patients over 65 years of age. The average age of patients with life-threatening complications was 9.6 years higher than those without (7). Adovica et al. describe a higher complication rate, ICU admission and LOS in older patients compared to younger patients, but without precise information on the age structure (18). In addition, Suehara et al. reported age as s significant variable associated with complications (72).

Furthermore, when looking at the subgroups, the incidence of ICU therapy and upper airway obstruction was higher in younger patients. In addition, Riekert et al. also described, that age was not significantly correlated with an ICU admission (75).

LOS is widely accepted as a surrogate marker of complicated course and adverse outcome in hospitalised patients (76, 77).

The mean LOS in our study was 3.92(3) ± 2.69 days, which was shorter than in other studies (10, 14, 66), but within the range of 3–10 days reported in previous studies (25, 30, 48, 68, 71, 78, 79). Park et al. reported a mean LOS of 12.43 days (44), Suehara et al. of 12.6 ± 14.4 days (72) and Seppänen et al. even reported a mean LOS of 14.8 days; however they only included 35 patients (14).

In our study, age as an isolated risk factor was associated with a longer LOS, but the difference was not significant (*p* = 0.129). Regression analysis showed a weak association between age and longer LOS (*p* = 0.001).

Wang et al. reported that older patients had a significantly longer LOS (80). Gams et al. described age as a significant predictor of longer LOS (66).

Park et al. identified age as a predictor for a LOS longer than 12 days. Among the other risk factors, age had the highest ratio for a longer LOS (44).

Regression analysis showed a significant association between peri/submandibular, parapharyngeal and oral base infections and prolonged LOS (*p* < 0.05). In older patients, the regression coefficient increased in all areas. As a result, we saw a stronger association between older patients with infections in these areas and prolonged LOS (*p* < 0.05). In contrast, Flynn at el. didn't find a significant association between prolonged LOS and involvement of the specific deep fascial spaces (67).

We also observed a significant association between lower molar infection and longer LOS, which also increased in the older population. In line with this, Alotaibi et al. described a longer LOS in patients with mandibular odontogenic infections compared to those with maxillary odontogenic infections (10).

In our study, regression analysis showed, that WBC and CRP levels on admission were positively correlated with LOS (*p* < 0.001 and complications (*p* < 0.001). Park et al. also described a CRP level > 10 mg/dl as a risk factor for prolonged LOS (44).

Flynn et al. described the WBC level as a significant predictor of reoperation and prolonged LOS (67). Mathew et al. reported a WBC count greater than 15 × 10^9^/L as a risk factor for life-threatening complications (11).

Many other studies have also shown that CRP levels can be a valuable marker for determining the severity of an odontogenic infections (9, 75, 76, 81–83). Sharma et al. reported a strong correlation between CRP levels and the severity of infection (81).

Ylijoki et al. and Riekert et al. reported a significantly higher WBC and CRP levels on admission in patients requiring intensive care compared to those not requiring intensive care (9, 75).

Riekert et al. conclude that higher CRP levels appear to be associated with the severity of deep cavity infections of odontogenic origin (75).

Regression analysis showed a significant correlation between higher WBC and CRP levels and complications as well as prolonged LOS, which is consistent with the findings of Gholami et al. (69). Wang et al. reported a CRP level > 100 µg/ml as a significant predictor of prolonged LOS, whereas a WBC level > 15,000/mm^3^ was not significantly associated with LOS (80). Consistent with this, Stathopoulos et al. also confirmed the CRP level as the only significant predictor of prolonged LOS.

Regarding the multivariate regression analysis, only higher CRP levels had a significant correlation with prolonged LOS (*p* < 0.001) and complications (*p* < 0.001). Therefore, we suggest that CRP is the most predictable laboratory marker of complicated courses in the elderly.

The three most frequency isolated bacteria by culture-based methods in this study were members of the *Streptococcus anginosus* group, *Prevotella* spp. and CNS, similar to the findings of Gams et al. (66) and other authors (78, 84).

Many studies have investigated the microbiology of odontogenic infections. Common to all these studies is the diversity of bacteria observed (22).

However, the literature is mixed regarding the predominant bacterial species in odontogenic infections (30, 78, 85). According to Gams et al., there are several possible explanations for this finding, including the laboratory culture technique, the protocol for transport to the laboratory and the method of sample collection (66). Previous reports show that standard culture methods of odontogenic infections yield an average of 2–8 species. When molecular techniques are used, the average is around 18 species (22, 86, 87). In addition, Walia et al. reported that the swab technique is associated with the isolation of gram-positive aerobes, whereas the aspiration technique results in more gram-negative anaerobes (30).

Furthermore, data from whole genome sequencing suggest that the role of aerobic gram-positive bacteria is overestimated when using culture-based diagnostics and that odontogenic infections are dominated by anaerobic *Prevotella*, *Porphyromonas* and *Fusobacterium* spp (88).

These limitations apply to our study as well. We used a swab technique followed by subsequent culture. Additionally, we had to group the results of cultures with no growth and those with a mixture of oral flora bacteria, where no further identification was conducted. This partly explains the relatively high percentage of cases (33%) in which no pathogenic bacteria were identified. Nevertheless, our findings regarding predominant bacteria are consistent with those of other authors using culture-based techniques (66, 78, 84).

*Candida* spp. was more frequently isolated from samples from elderly patients than from younger patients, whereas viridans group streptococci were more frequently identified in non-elderly patients. *Candida* spp. and viridans group streptococci are not known to be the predominant species in odontogenic infections. Therefore, the absence of viridans group streptococci, which are part of the normal oral flora, and a higher abundance of *Candida* spp. in the elderly may be a surrogate for an altered composition of the oral microbiome in the elderly patients included in this study. The prevalence of *Candida* spp. carriage has previously been shown to increase with age (89). This could be induced by previous antimicrobial therapy or denture prosthesis, which could explain the higher prevalence of *Candida* spp. in the elderly (90, 91).

The study has a few limitations, and some study parameters are debatable:

First, the study is a monocentric, retrospective study, all patients came from the catchment area of the Klinikum Oldenburg AöR, which represents a subpopulation in Germany.

To ensure better comparability of the study groups and to avoid possible alterations in the results, we defined a lot of stringent exclusion criteria. Nevertheless, these facts could influence the results in the sense of a selection bias and can limit the transferability of the results to other countries and ethnic groups.

Referring to the joint statement of the significant German Geriatric Societies we defined 70 years as cutoff age for elderly patients. Referring to the literature, some authors prefer 65 years as cutoff age (41, 63) again other authors use 60 years for cutoff definition (1). Nevertheless, there is no generally cutoff value for the calendared age and the resulting is a “grey area” regarding the allocation of the age group between 60 and 70 years. Despite this problem to define geriatric patients based on their calendar age, other important factors like multimorbidity, frailty and decrease of functional reserve had to be considered for the definition of the geriatric patient. An improvement for further studies could be the selection by a geriatric assessment tool.

## Conclusions

The data from this study show, that age alone is not a risk factor for complications and longer LOS. However, in combination with specific anatomical spaces, in particular the parapharyngeal and oral base space, older patients have a higher likelihood of complications and longer LOS.

In addition, we found a significant effect of CRF on complications and LOS with an increase especially in the older population.

The present study shows that high WBC and especially high CRP levels on admission can serve as sensitive and reliable predictive markers for complications and prolonged LOS. Furthermore, in older patients, CRP is the more predictable marker of a complicated course and prolonged LOS.

## Data Availability

The original contributions presented in the study are included in the article/Supplementary Material, further inquiries can be directed to the corresponding author/s.
