# Supplementary material for: Outcome predictors of odontogenic abscesses in the elderly
Source: Front Oral Health. 2024 Dec 2;5:1486182. doi: 10.3389/froh.2024.1486182 (PMC11646891; doi:10.3389/froh.2024.1486182)
Supplement: Supplementary file 1 [file Datasheet1.pdf]

<u>**Supplementary material**</u>

Figures

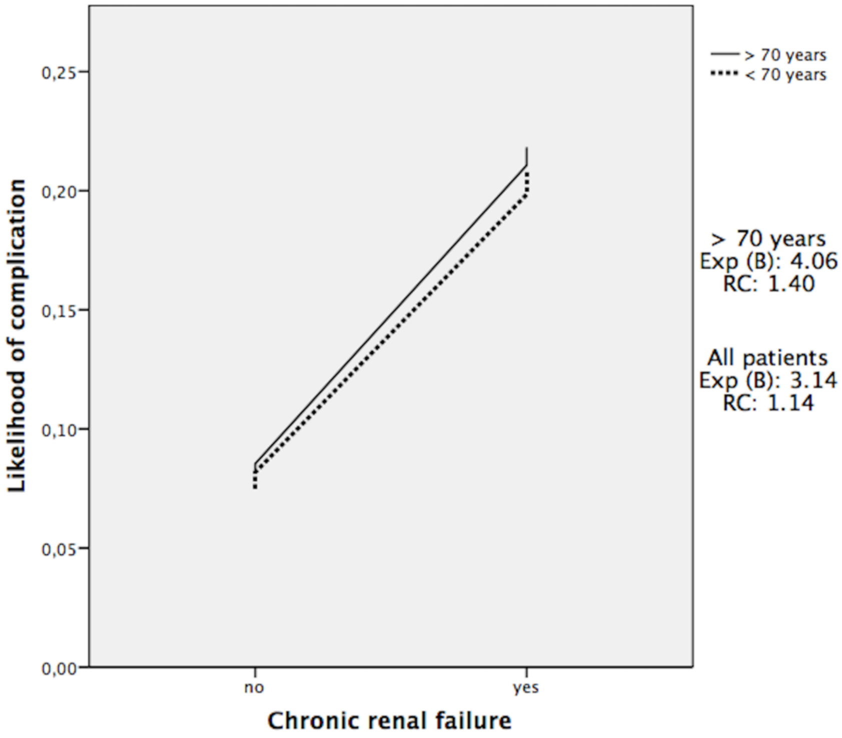

Figure 1. Effect plot of complications depending on pre–existing CRF

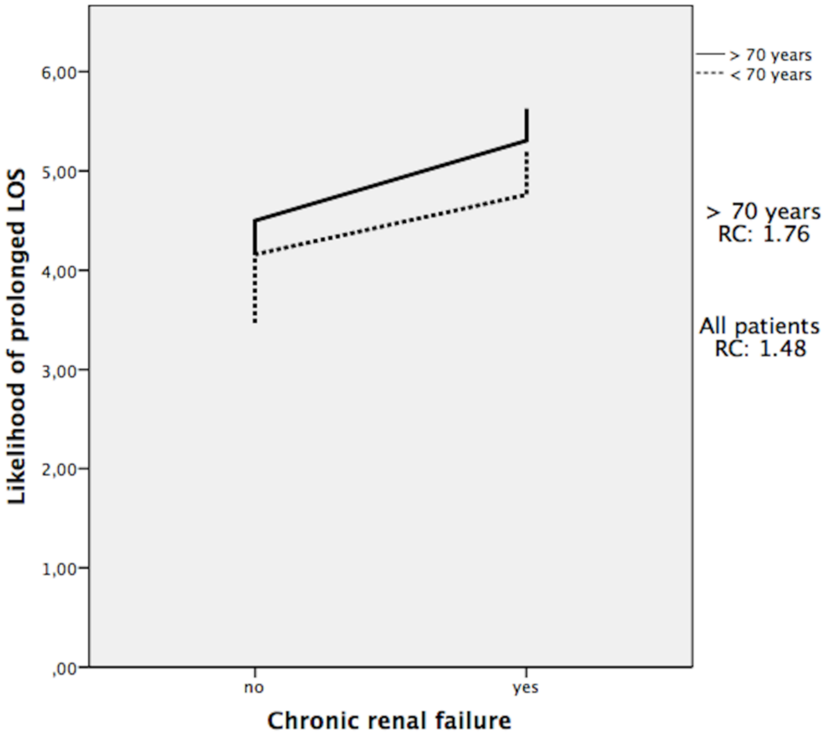

Figure 2. Effect plot of LOS depending on pre-existing CRF

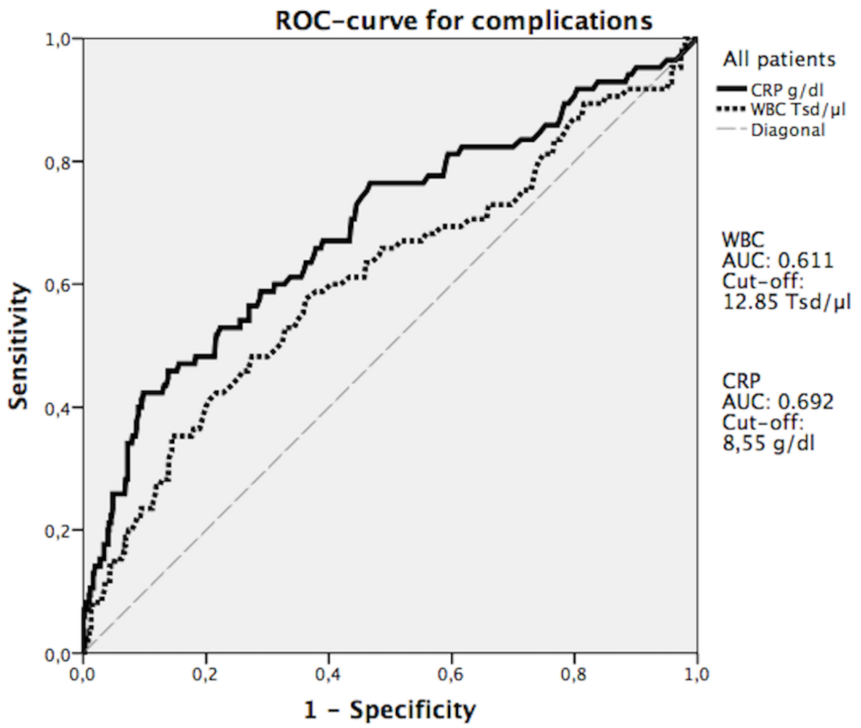

Figure 3. ROC – analysis for CRP and WBC (all patients)

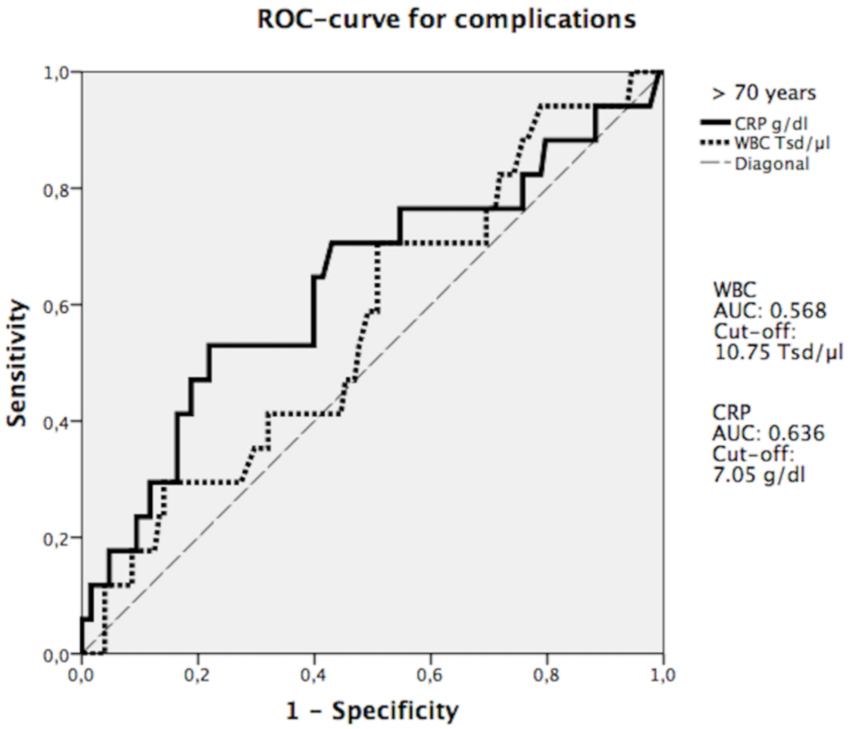

Figure 4. ROC-analysis for CRP and WBC (patients >= 70 years)

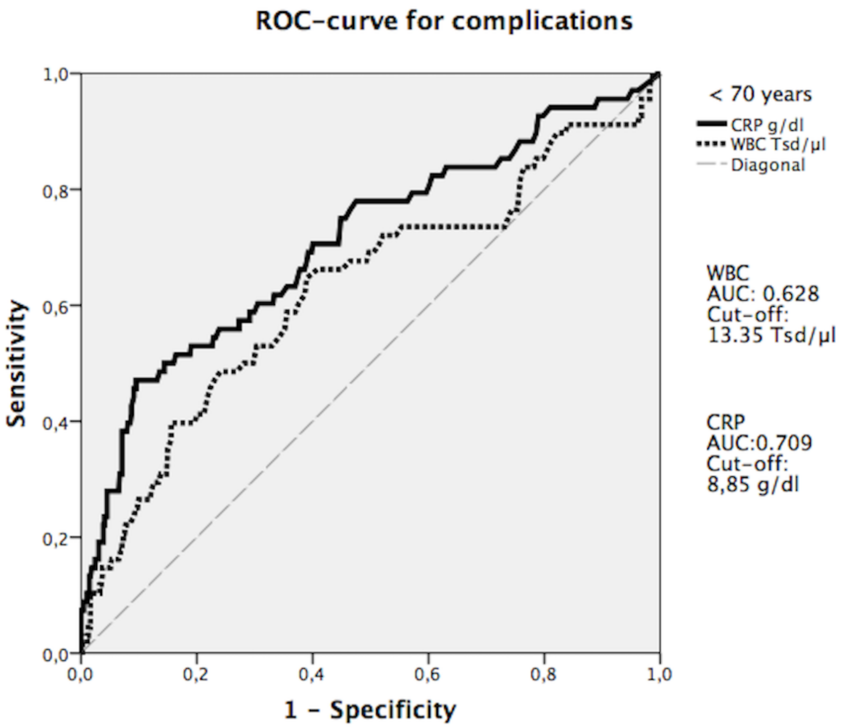

Figure 5. ROC-analysis for CRP and WBC (patients < 70 years)
